# Comprehensive Radiomics Analysis to Enhance Breast Lesion Characterization Using QUS Spectral Parametric Imaging

**DOI:** 10.3390/cancers18162548

**Published:** 2026-08-08

**Authors:** Laurentius Oscar Osapoetra, Lakshmanan Sannachi, Schontal Halstead, David Alberico, Daniel Moore-Palhares, Gregory J. Czarnota

**Affiliations:** 1 Physical Sciences, Sunnybrook Research Institute, Sunnybrook Health Sciences Centre, Toronto, ON M4N 3M5, Canada; laurentiusoscar.osapoetra@sunnybrook.ca (L.O.O.); lakshmanan.sannachi@sunnybrook.ca (L.S.); schontal.halstead@gmail.com (S.H.); david.alberico@sri.utoronto.ca (D.A.); moorefreit@hhsc.ca (D.M.-P.); 2 Department of Radiation Oncology, Odette Cancer Centre, Sunnybrook Health Sciences Centre, Toronto, ON M4N 3M5, Canada; 3 Department of Radiation Oncology, University of Toronto, Toronto, ON M5T 1P5, Canada; 4 Department of Medical Biophysics, University of Toronto, Toronto, ON M5G 1L7, Canada

**Keywords:** quantitative US, breast cancer, radiomics, predictive analytics, machine learning

## Abstract

**Background/Objectives:** We aimed to evaluate the generalizability of QUS spectral parametric imaging radiomics for breast tumor characterization in a large cohort through methodological optimization and rigorous validation, including comprehensive feature extraction and evaluation of multiple classifiers, addressing limitations of previous studies potentially affected by data leakage. **Methods:** This study included 277 participants (185 malignant cases (median age, 51 years [IQR: 44–63 years]) and 92 benign cases (median age, 46 years [IQR: 38–51 years]) with breast masses, acquired between September 2014 and October 2021. QUS spectroscopic analysis resulted in five maps, from which first-order statistical, various textural, and morphological features were extracted from both the tumor core and a surrounding 5 mm margin. The ground truth label was determined from histopathological analysis. Predictive models were developed to distinguish malignant from benign lesions. Their generalization performance was assessed using hold-out validation. Beyond assessing models’ performance, SHapley Additive exPlanations (SHAP) analysis identified the most influential features to the predictions. **Results:** 329 radiomics features demonstrated statistically significant differences (*p*-values < 0.00005). Averaged across 50 partitions, SVM-Linear models produced test performance of 82 ± 8% (CI: 59–97) recall, 79 ± 9% (CI: 50–100) specificity, and 0.87 ± 0.05 (CI: 0.71–0.98) area under the receiver operating characteristic curve (AUROC). The best single partition performance with an SVM-Linear model was 92% recall, 89% specificity, and 0.97 AUROC. **Conclusions:** This work establishes a performance benchmark for handcrafted radiomic features derived from QUS spectral parametric images in breast lesion characterization, demonstrating its strong generalization and servicing as a reference for future model development.

## Simple Summary

A multivariate quantitative radiomics model of quantitative ultrasound (QUS) spectral parametric images demonstrated strong generalization performance in distinguishing malignant from benign breast lesions. In participants with breast lesions, 329 radiomics features demonstrated statistically significant differences (*p*-values < 0.00005) between malignant and benign groups. An SVM-Linear model achieved the best performance of 92% recall, 89% specificity, and an area under the receiver operating characteristic curve of 0.97 on an independent test set. The model produced the average classification performance of a recall of 82 ± 8% (CI: 59–97), with a specificity of 79 ± 9% (CI: 50–100), and an area under the receiver operating characteristic curve of 0.87 ± 0.05 (CI: 0.71–0.98) on the hidden test sets.

## 1. Introduction

Breast cancer is the most frequently diagnosed cancer in women and ranks as the second leading cause of cancer-related deaths amongst women [1]. In the year 2020, the United States witnessed 239,612 new cases of female breast cancer, with a regrettable 42,273 women losing their lives to this disease [2]. The significance of early detection cannot be overstated, as it leads to improved overall prognoses and allows for the administration of more effective treatments, especially when the disease is identified in its early stages. Achieving this goal necessitates the employment of accurate and precise diagnostic techniques.

Breast cancer screening usually starts with mammography, followed by B-mode US, dynamic contrast-enhanced MRI (DCE-MRI) when necessary, and core-needle biopsy. Mammography, though widely available, has limitations, especially in dense breast tissue, leading to false negatives [3,4]. Furthermore, interpretation variability in mammograms has led to high rates of false positives and false negatives [5]. Biopsy is the gold standard for tumor diagnosis [4], but is invasive, painful, and carries a theoretical risk of tumor cell migration. Due to the low specificity of B-mode US [3] and MRI, unnecessary biopsies are common [4]. While DCE-MRI improves specificity [6], it has longer wait times and limited availability. Thus, there is a critical need for rapid, accurate imaging methods to characterize breast lesions, facilitating early breast cancer detection and patient triage.

Previous studies have used B-mode US features to characterize breast lesions [7,8,9]. While useful for visualizing anatomy, B-mode images lack quantitative information about tissue microstructures, which can lead to inter-observer variability. Additionally, they are susceptible to variations due to imaging system settings and operator technique, limiting their reliability for diagnostic and prognostic use. Quantitative US (QUS) techniques address the limitations of traditional B-mode imaging by extracting tissue microstructural features with potential diagnostic and prognostic value. QUS spectroscopy analyzes the spectral content of US radiofrequency (RF) data to assess acoustic backscattering properties and has been applied for characterizing tumors [10,11,12,13,14], therapy monitoring [15,16,17,18], and detecting tumor deposits in ex vivo lymph nodes [19]. QUS echo envelope analysis evaluates the spatial organization of acoustic scatterers via statistical analysis of US RF envelopes [20,21]. Additionally, elasticity imaging methods like shear wave elastography (SWE) provide macro-elastic parameters, such as shear modulus, for tissue characterization [13].

Tumor microenvironment, physiology and metabolism exhibit heterogeneities that carry significant diagnostic and prognostic implications in cancer characterization [22,23,24,25]. We postulated that a comprehensive radiomics approach to QUS spectral parametric images of benign and malignant breast lesions could facilitate the development of a robust multivariate classification model. In contrast to previous works that built models exclusively based on GLCM features [10,11], or devised models for GLCM, GLRLM, and GLSZM separately [12], here we utilized a comprehensive textural feature space.

This study offers several key contributions, notably in advancing model development and evaluation strategies, with a strong emphasis on minimizing data leakage to reduce bias in classification performance. We standardized feature extraction using the PyRadiomics library [26] and implemented comprehensive preprocessing steps (such as skew correction, feature standardization, and outlier detection) to establish a robust predictive analytics framework. The inclusion of a larger patient cohort, a more comprehensive set of radiomics features, and a broader range of machine learning classifiers supported the development of a more generalizable model. The model’s performance was rigorously assessed using multiple stratified train–validation–test splits, ensuring an objective evaluation of generalization capability. Beyond traditional classification metrics, we enhanced model interpretability through SHapley Additive exPlanations (SHAP) analysis [27] to identify the most influential features driving model predictions. Collectively, these contributions establish a new performance benchmark for breast lesion characterization using QUS spectral parametric imaging and radiomics.

## 2. Materials and Methods

### 2.1. Participant Selection

This study received approval from the Sunnybrook research ethics board (SUN-2094) and was carried out at a single institution. It was registered on clinicaltrials.gov (NCT04050423) and conducted in accordance with good clinical practice guidelines and the Declaration of Helsinki. All eligible participants provided written informed consent prior to enrollment. Participants included individuals with breast lesions detected on US following initial identification through clinical assessment or mammography. Exclusion criteria consisted of a previous diagnosis of breast cancer (*n* = 8), dropout (*n* = 2), lesions that were not detectable on US imaging and issues in ROI detection (*n* = 27), no volumetric RF and missing data (*n* = 16), wrong acquisition settings (*n* = 3), and label issues (*n* = 1). Recruitment took place between September 2014 and October 2021. The final study cohort included 277 participants with breast lesions (from the original cohort of 334 participants), comprising 92 benign and 185 malignant cases. A portion of the participants had been included in previous studies: 78 [10], 204 [11], and 204 [12]. The current study utilized a comprehensive set of radiomics features and implemented state-of-the-art model building and evaluation strategies.

The classification of benign and malignant tumors was determined using clinical patient reports, which included assessment by radiologists using US B-mode and MR images, along with histopathological findings for lesions subjected to biopsies. Some lesions from the benign group were from participants that were solely subjected to follow-up imaging surveillance, as biopsies were considered unnecessary. For all these participants, clinical patient history indicated that the associated benign lesions had remained stable for an average of ten years.

We compared our radiomics approach to the breast imaging reporting and data system (BI-RADS) score [28], obtained from blinded radiologist assessments. Lesions with BI-RADS ≤ 3 were considered benign, and those with BI-RADS ≥ 4 were considered malignant, following the cut-off used in previous studies.

### 2.2. Data Acquisition

We acquired US RF data using a Sonix Touch US system (Ultrasonix Medical Corp., Richmond, BC, Canada) equipped with a linear-array transducer (L14-5/60W) operating at a center frequency of 6.5 MHz with a bandwidth of 3–8 MHz. The system specifications are summarized in Table A1.

The scans were performed by a radiologist or sonographer with expertise in breast US imaging. Tumors, along with their 5 mm tumor rims, were manually delineated. Multiple RF frames were collected throughout the three-dimensional tumor volume, capturing successive tumor slices. Parametric maps were then derived from the defined regions of interest (ROIs), as described below, and radiomics features were subsequently extracted from these maps.

### 2.3. QUS Spectral Parametric Imaging

Alongside standard B-mode images of tumors, QUS spectral parametric images were generated from both the tumors and their surrounding 5 mm margins. These QUS spectral parameters were obtained through spectral analysis of windowed US RF data. We utilized a 2 mm by 2 mm sliding window with a 94% window overlap in both the range and lateral directions. This window size was chosen to include an adequate number of acoustic wavelengths for reliable spectrum estimation while maintaining enough spatial resolution to resolve regions of differing microstructural characteristics [29]. This window was systematically shifted across the entire ROI to capture each pixel in the resulting parametric images.

The mean spectrum was estimated from each RF data block, with a Hanning gating function applied along the axial direction of the RF signal. The signal was then transformed into the frequency domain using a fast Fourier transform (FFT). Subsequently, the power spectra were averaged across all lateral directions (columns within the RF block) to obtain a single representative spectrum. Additionally, attenuation correction was applied to correct for US loss as it propagated through intervening tissues and the tumor. A fixed attenuation coefficient value was assumed for the skin layer, while the tumor attenuation coefficient was estimated using a spectral difference method [30].

Spectral parameters were extracted from parametrization of the average power spectra. We derived linear-fit spectral parameters, including mid-band fit (MBF), spectral slope (SS), and 0 MHz spectral intercept (SI). The MBF and SI are indicators of the amount of acoustic backscattering, whereas SS is associated with the effective size of acoustic scatterers [29]. Furthermore, fitting an acoustic scattering model to the measured backscatter coefficient (BSC) allowed the estimation of acoustic scatterer parameters including average/effective acoustic concentration (AAC/EAC) and average/effective scatterer diameter (ASD/ESD) parameters [29].

We performed the analysis for all points within the ROI, resulting in parametric images that portrayed the spatial distributions of QUS spectral parameters. Next, we derived a comprehensive set of radiomics features related to first-order statistics of pixels, texture, and morphology from these images.

### 2.4. Feature Engineering

We extracted handcrafted radiomics features from the parametric images of the breast tumor and its 5 mm tumor rim. We utilized the PyRadiomics (v. 3.1.0) package to obtain these features [26], allowing for standardization of the process. The parametric map voxels were resampled into regular grids using a B-Spline interpolator of order 3 to preserve texture and smoothness, prior to feature extraction. The gray values of the parametric maps were discretized into a fixed number of levels (i.e., bin count) of 32. We obtained a total of 939 features from the tumor core and its margin. These comprise first-order statistical features (*n* = 18), shape (*n* = 9), and textural features. Textural features include the Gray-level co-occurrence matrix (GLCM) features (*n* = 24) [31], gray-level run-length matrix (GRLM) features (*n* = 16) [32,33,34,35], gray-level size-zone matrix (GLSZM) features (*n* = 16) [36], neighboring gray tone difference matrix (NGTDM) features (*n* = 5) [37], and gray-level dependence matrix (GLDM) features (*n* = 14) [38]. The different features extracted are tabulated in Table A2.

Vectors of radiomics features were obtained from parametric maps and subsequently averaged based on the ROI size to obtain the weighted average vector of radiomics features from each participant. The data matrix is formed by these vectors of radiomics features.

### 2.5. Data Preprocessing

#### 2.5.1. Data Partitioning

A stratified random split on the whole dataset was performed, partitioning it into an 80% development set and a 20% test set. The development set was used for model building and selection. The development set was further split into *K* = 5 approximately equal-sized parts, maintaining the original proportion of the two classes. For the *k*-th part, the model was fit to the other *K*-1 parts and the classification performance of the fitted model when predicting the *k*-th part of the development set was calculated. The classification performance from the *K* cross-validation (CV) folds was averaged. This partition was used for selecting the optimum set of features, optimizing the hyperparameters of the classifier, and evaluating the validation performance. This random split was repeated fifty times, and the average classification performance was reported.

#### 2.5.2. Standardization and Outlier Identification

First, any skewness in the development data was identified. For normally distributed features, the data was standardized by transforming each feature to have a zero mean and unit variance. On the other hand, for non-normally distributed features, a non-parametric quantile transformation was applied to transform each feature into a normal distribution. Subsequently, outliers were removed from the development set using an isolation forest technique [39].

#### 2.5.3. Feature Selection/Dimension Reduction

The data reduction phase encompassed both feature reduction and data balancing. A two-step univariate feature selection process was utilized, employing filter methods. In the initial step, a hypothesis test was conducted for each feature to assess whether the means were significantly different.

In the second stage, a filter-based feature selection method was implemented using the minimal-redundancy–maximal-relevance criterion (MRMR) [40]. The MRMR identifies a subset of features (*n* = 150) with the highest relevance to the target class, assessed by the mutual information (MI) metric, while minimizing correlation among them [40]. We utilized the MRMR function from the Pymrmre software library (v. 1.0.7).

Due to the univariate nature of filter-based methods for feature selection, a wrapper method was subsequently applied to capture the interaction between features. A forward sequential feature selection (SFS) was conducted to identify an optimal feature set. Features were added based on their performance across *K* = 5 CV folds, selecting those that contributed to the best average performance. A model complexity analysis was conducted by varying the number of selected features including 4, 5, 6, 7, 8, 9, 10, 15, and 20 features. Prior to performing SFS, the reduced dataset was balanced using a synthetic minority oversampling technique (SMOTE) [41]. The SMOTE addresses class imbalance by oversampling the minority class through the creation of synthetic samples along line segments connecting any or all the *k* nearest neighbors from the minority class [41]. Additionally, we provided a sensitivity analysis for handling data imbalance using random undersampling. We utilized the SMOTE and the random undersampling functionalities from the Imbalanced-learn software package (v. 0.14.1).

### 2.6. Model Building and Evaluation

Multivariate predictive analytic models were developed to distinguish malignant from benign breast lesions. On the development set, various machine learning classifiers were trained on the training set and their performance was evaluated on the validation set. CV was employed to select the optimal model, including its hyperparameters. The finally selected model was trained on the whole development set and then utilized to predict a hidden test set. These classifiers encompassed linear discriminant analysis (LDA), *k*-nearest neighbors (KNNs), linear support vector machines (SVM-Linear), SVM-RBF [42], AdaBoost [43], gradient boosting [44], and a shallow multilayer perceptron (MLP) [45]. Table A3 provides details on the hyperparameters of the classifiers.

**Figure 1:**
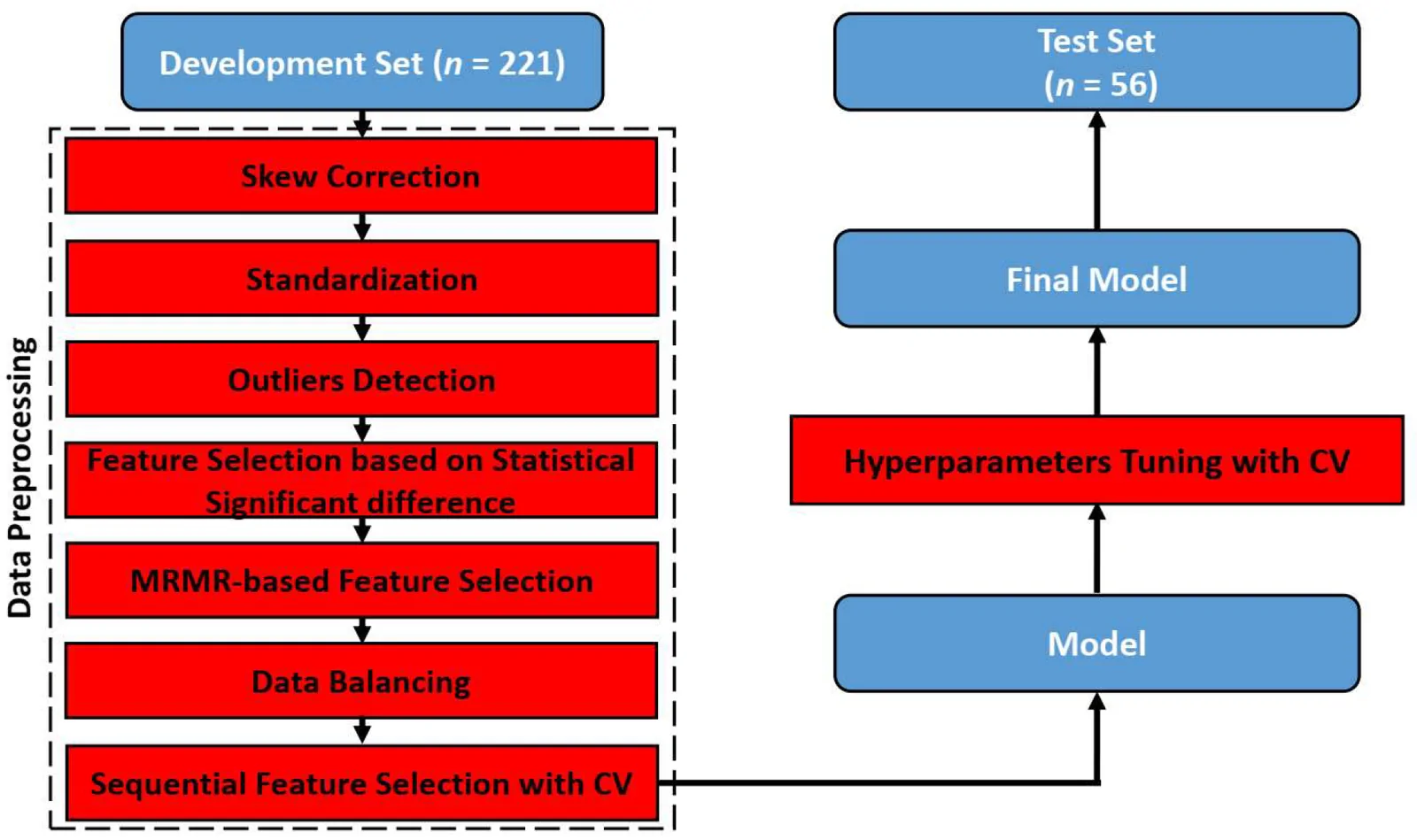
Methodology for data preprocessing and model development workflow for QUS spectral parametric imaging radiomics. Datasets and models are represented by blue rectangles with rounded corners, while processing steps are represented by red rectangles. Arrows indicate the direction of the process. The entire dataset was partitioned into development and test sets before further analysis. Employing a stratified random split, we ensured the preservation of the original proportion between malignant and benign groups. The data preprocessing steps encompass skew correction, feature standardization, outlier removal, univariate feature selection based on statistically significant differences, MRMR-based univariate feature selection, data balancing using either SMOTE or RUS, and wrapper-based feature selection utilizing a forward SFS. SFS was implemented using a stratified five-fold CV with three repetitions. This process yielded the model, which is a combination of *p* features, where *p* varied from 4 to 20 features. Subsequently, we fitted machine learning classifiers on the selected features and optimized their hyperparameters. We employed a stratified five-fold CV with three repetitions to select the optimal set of hyperparameters. Finally, the resulting final model underwent testing against a hold-out test set. The entire process was repeated fifty times, each with a different stratified random splitting, to acquire statistical measures of classification performance. MRMR: maximal relevance minimal redundancy. SMOTE: synthetic minority oversampling technique. RUS: random undersampling. SFS: sequential feature selection. CV: cross-validation.

We reported classification metrics on both the validation and test sets. The metrics include recall, specificity, accuracy, balanced accuracy, precision, negative predictive value (NPV), F1-Score, area under the receiver operating characteristics curve (AUC), and area under the precision-recall curve (AUPRC). We reported classification performance that includes the best result from a single partition and the average results from fifty partitions. Figure 1 depicts the data processing and model evaluation workflow. We executed this workflow using the Scikit-learn software package (v. 1.7.2). We provide the metrics’ definitions as follows:

$$Recall=\frac{TP}{TP+FN}$$

$$Specificity=\frac{TN}{TN+FP}$$

$$Accuracy=\frac{TP+TN}{TP+FN+TN+FP}$$

$$Balanced\;Accuracy=\frac{1}{2}\left(Recall+Specificity\right)$$

$$Precision=\;\frac{TP}{TP+FP}$$

$$NPV=\frac{TN}{TN+FN}$$

$$F1-Score=\frac{2\;\times \;Precision\;\times \;Recall}{Precision+Recall}$$

where TP = True Positives, FN = false negatives, TN = True Negatives, and FP = false positives. The 95% confidence interval (CI) was estimated for each metric by bootstrapping the hold-out test scores from fifty partitions. We resampled the test scores 1000 times for a single partition. In addition, AUROC differences were reported for paired AUROCs from the best model with the other classifiers, where the average AUROC differences and their 95% confidence interval (CI) were estimated through bootstrapping (*n* = 1000). These are provided in the Table A6A,B. Appendix B provides the computational complexity analysis for the proposed framework.

### 2.7. Statistical Analysis

We conducted statistical analysis utilizing the SciPy software package (v. 1.15.3). The test for any statistically significant difference was performed using either a two-sample *t*-test or a Mann–Whitney U-test, depending on the data distribution. We used the Shapiro–Wilk normality test to check for normality. A Bonferroni correction was applied to adjust the significance threshold to *α* = 0.05/(# of hypothesis tests), to counteract the elevated risk of false positives due to multiple hypothesis tests. As a result, we utilized an adjusted significance level of *α* = 0.05/939 = 0.00005 for each feature.

## 3. Results

### 3.1. Patient Characteristics

This study enrolled a total of 277 breast lesion participants, with 185 malignant cases (median age, 51 years [IQR: 44–63 years]) and 92 benign cases (median age, 46 years [IQR: 38–51 years]). Table 1 summarizes the clinical characteristics, with more detailed per-patient characteristics provided in Table A4 and Table A5 for the benign and malignant groups, respectively. The malignant groups included IDC (*n* = 167), ILC (*n* = 9), and other malignant lesions (*n* = 9). The benign groups comprised FA (*n* = 50), simple cyst (*n* = 20), complicated cyst (*n* = 11), and other benign lesions (*n* = 11). These sub-types were obtained from clinical patient records that included histological findings through biopsied samples and radiological results. For the malignant sub-cohort, hormone receptor statuses—estrogen receptor (ER), progesterone receptor (PR), and human epidermal growth factor receptor 2 (HER2)—are provided in Table A5.

### 3.2. QUS Spectral Parametric Images

**Figure 2:**
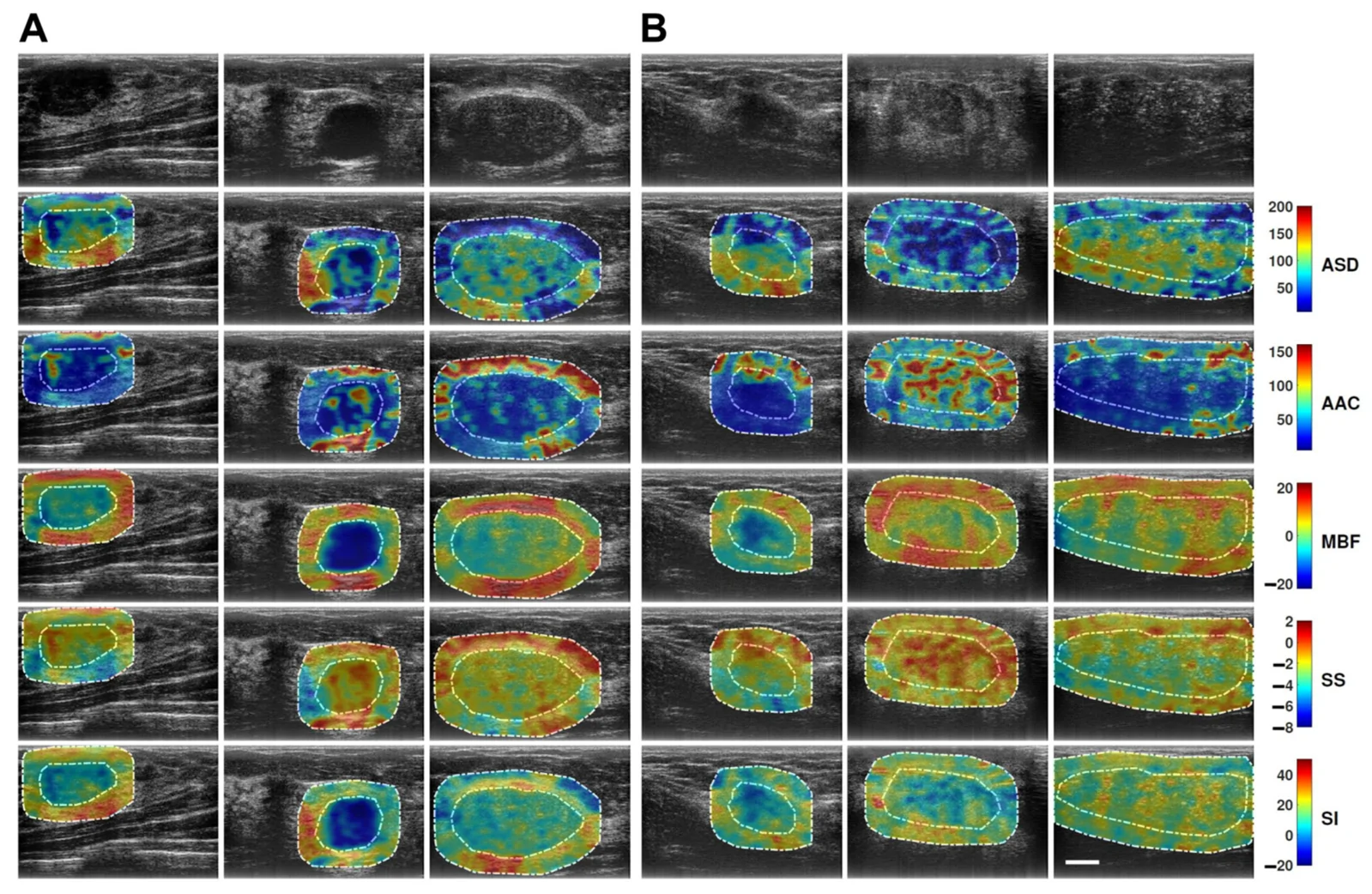
Representative B-mode and QUS spectral parametric images of ASD, AAC, MBF, SS, and SI for (**A**) benign (left three columns) and (**B**) malignant (right three columns) breast lesions. These correspond to the full FOV of 4 cm axially and 6 cm laterally. The color bar ranges are 195 µm for ASD, 155 dB/cm^3^ for AAC, 44 dB for MBF, 10 dB/MHz for SS, and 70 dB for SI. The scale bar represents 1 cm. The benign lesions were diagnosed as fibroadenoma, simple cyst, and fibroadenoma or phyllodes tumor. The malignant lesions were diagnosed as IDC, ILC, and DCIS. From these maps, a comprehensive set of radiomics features, including basic statistical, various textural, and morphological features, was extracted from the tumor core and its 5 mm tumor margin. These are utilized to build predictive models to differentiate malignant from benign breast lesions. ASD: Average scattering diameter. AAC: average acoustic concentration. MBF: mid-band fit. SS: spectral slope. SI: spectral intercept. FOV: field-of-view. IDC: invasive ductal carcinoma. ILC: invasive lobular carcinoma. DCIS: ductal carcinoma in situ.

Figure 2 shows representative B-mode and spectral parametric images of ASD, AAC, MBF, S, and SI. The representative benign cases were FA, simple cyst, and FA/phyllodes tumors. The representative malignant cases were IDC, ILC, and DCIS. All masses in the breast appeared typically hypoechoic, with cystic structures demonstrating notable hypo-echogenicity.

### 3.3. Radiomics Features

**Figure 3:**
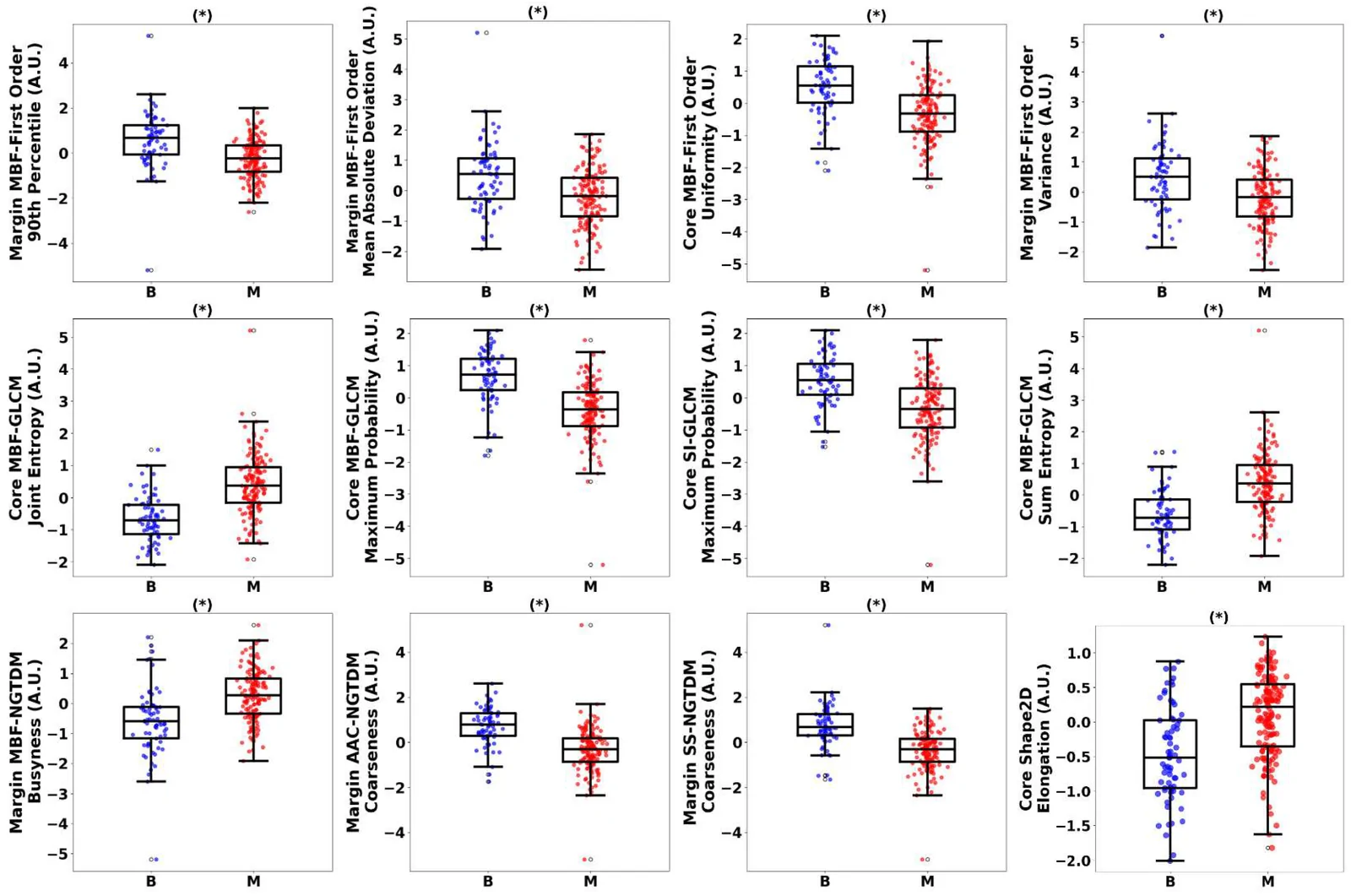
Box and scatter plots of representative discriminative features (*p*-values < 0.00005) between benign (‘**B**’) and malignant (‘**M**’) groups, with statisitically significant features indicated by the asterisks (*). Benign samples are represented by blue circles, while malignant samples are represented by red circles. These features encompass basic statistical, various textural, and morphological features, with the majority being textural and first-order statistical features from the MBF maps. The features were transformed into standard normal distribution using the *z*-transformation and quantile transformation for non-skewed and skewed features, respectively. MBF: mid-band fit. AAC: average acoustic concentration. SS: spectral slope. GLCM: gray-level co-occurrence matrix. NGTDM: neighboring gray tone difference matrix.

In total, 329 radiomics features were found to demonstrate statistically significant differences (*p*-values < 0.00005) between benign and malignant groups. Figure 3 depicts box and scatter plots representative of these discriminating features. The bottom and top edges of the box indicate the 25th and 75th percentiles, respectively, while the central mark in each box represents the median. The whiskers are 1.5 times the interquartile range. Features which were the most different included Core MBF-GLCM Joint Entropy, Margin MBF-GLSZM Gray-Level Nonuniformity, Core MBF-GLCM Joint Energy, Margin SS-NGTDM Coarseness, and Core SI-GLCM Joint Entropy. On the other hand, features such as Margin MBF-GLCM Contrast, Core SI-GLSZM High Gray-Level Zone Emphasis, Core MBF-NGTDM Busyness, and Margin SS First-Order Mean did not demonstrate statistically significant differences (*p*-values > 0.00005).

### 3.4. Classification Results

For each classifier, we selected the final model based on the highest average validation F1-Score. Model complexity was systematically varied by adjusting the number of selected features from four to 20. Specifically, for each data partition, we selected model complexity based on its F1-Score classification performance on the validation set. Table 2 presents a comprehensive summary of the final models, demonstrating their robust classification performance on hidden test datasets. The test sets comprised 56 participants.

Within the set of machine learning classifiers applied to this specific dataset, linear models such as LDA and SVM-Linear classifiers exhibited the most effective generalization performance in terms of accuracy and balanced accuracy scores. Across the partitions, the SVM-Linear models achieved a recall of 82 ± 8%, specificity of 79 ± 9%, balanced accuracy of 80 ± 5%, and AUROC of 0.87 ± 0.05 on the hold-out test sets. Similarly, the LDA models attained a recall of 80 ± 8%, specificity of 78 ± 8%, balanced accuracy of 79 ± 5%, and AUROC of 0.87 ± 0.05 on the hold-out test sets.

The Kruskal–Wallis H-test yielded a test statistic of 93.93, surpassing the critical value of chi-squared with *g* − 1 degrees of freedom at the desired significance level alpha set to 0.05. Consequently, the data supports the alternative hypothesis that the balanced accuracies differ among the various classifiers (*p* < 0.05). Post hoc tests using Dunn’s test [46] indicated no statistically significant difference in performance between LDA and SVM-Linear. Both linear classifiers significantly outperformed (*p* < 0.05) the nearest neighbor, SVM-RBF, boosting techniques, and the shallow ANN.

Across fifty stratified random partitions of the whole dataset, there is a variation in the number of selected features that leads to the maximum validation F1-Score. On average, the 13-feature model led to the best validation performance. Table 3 tabulates the optimum features set for the 13-feature LDA and SVM-Linear models. The optimal features were those mostly selected for building a model across fifty random partitions of the whole dataset. Among the most selected features for the SVM-Linear model, the majority were textural and first-order statistical features from MBF parametric maps. These predominantly included features such as GLCM Maximum Probability, GLSZM Large-Area Low Gray-Level Emphasis, First Order 90th Percentile, First-Order Mean Absolute Deviation, and GLCM Joint Entropy.

### 3.5. Sensitivity Analysis of Approaches to Data Imbalances

We compared the implemented data-centric approach for handling class imbalance using SMOTE with the corresponding random undersampling (RUS) approach. For RUS, we randomly undersampled the majority malignant group in the development set. Table 4 presents the generalization performance of the thirteen-feature models based on the RUS approach across fifty stratified random partitions. Across these partitions, the LDA model achieved a recall of 81 ± 7%, specificity of 80 ± 9%, balanced accuracy of 81 ± 6%, AUROC of 0.87 ± 0.06, and AUPRC of 0.93 ± 0.05. SVMs showed similar performance: the SVM-Linear model obtained a recall of 79 ± 8%, specificity of 80 ± 9%, balanced accuracy of 79 ± 6%, AUROC of 0.87 ± 0.05, and AUPRC of 0.92 ± 0.04, while the SVM-RBF model achieved a recall of 80 ± 8%, specificity of 82 ± 8%, balanced accuracy of 81 ± 5%, AUROC of 0.87 ± 0.05, and AUPRC of 0.93 ± 0.03.

### 3.6. Comparison with BI-RADS

Table 5 provides a comparison of classification performances between QUS radiomics and machine learning versus the thresholding of the BI-RADS scores for the sub-cohort of suspicious breast lesions. Across the partitions, the averaged classification performance based on the BI-RADS score was a recall of 98 ± 5%, specificity of 39 ± 26%, and balanced accuracy of 68 ± 13%. QUS radiomics with linear SVM classifier obtained a recall of 74 ± 18%, specificity of 78 ± 19%, and balanced accuracy of 76 ± 12%. We utilized a t-test to compare the means of classification metrics between the BI-RADS and the QUS radiomics + machine learning. The specificity and balanced accuracy scores obtained with the QUS radiomics and machine learning framework were statistically significantly higher than those from thresholding the BI-RADS scores (*p*-values < 0.05). Still, the BI-RADS as a clinical feature still outperformed radiomics framework in terms of detection of malignant lesions.

### 3.7. Explainability Analysis

**Figure 4:**
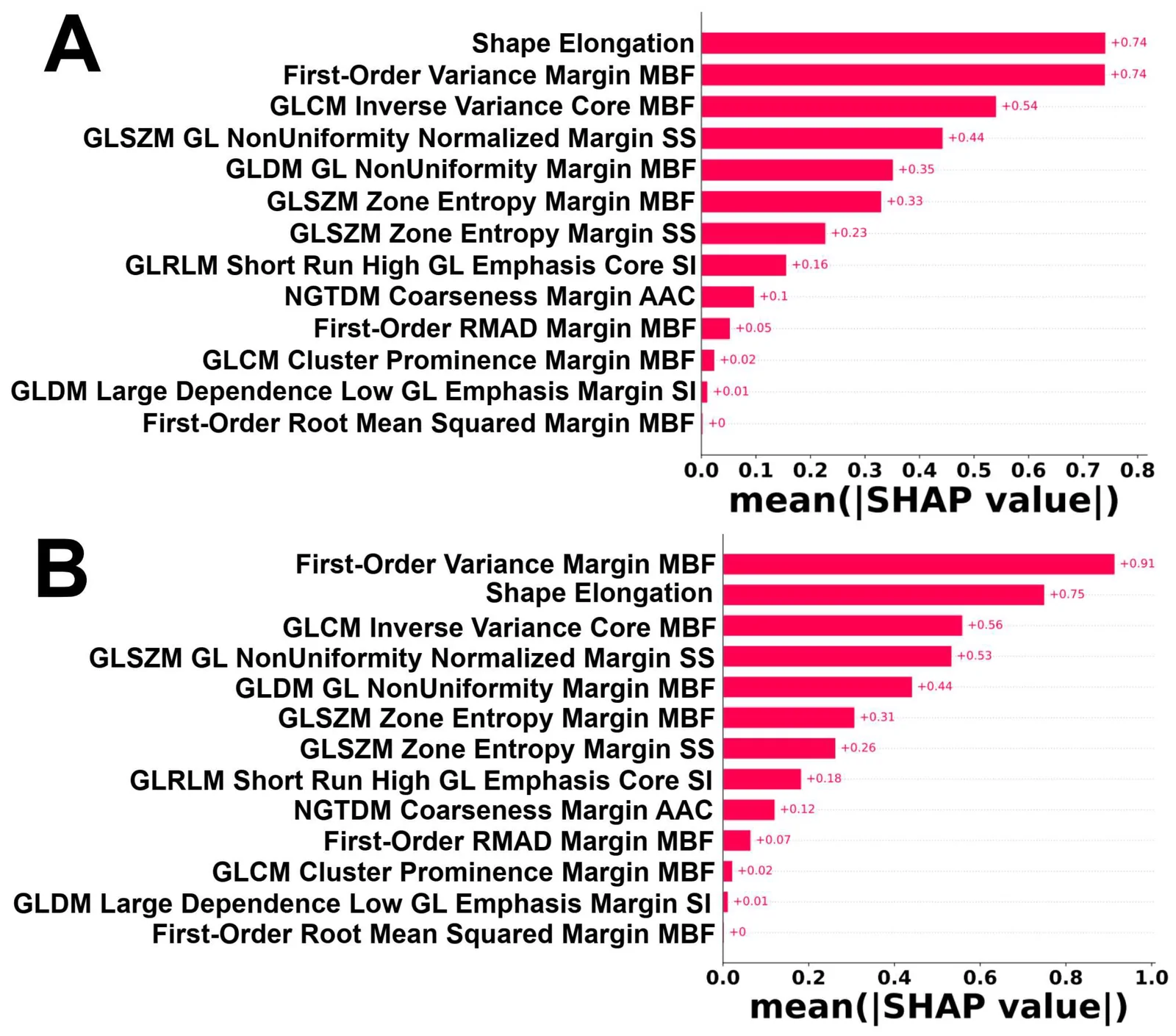
Model interpretability analysis. Representative SHAP values from (**A**) a validation set and (**B**) test set. SHAP analysis determined that the first-order statistical variance feature from the margin MBF map as the most influential to the predictions. This was followed by the 2D shape elongation and the GLCM inverse variance from the core MBF map. These three features were consistently identified as the most important across the five CV folds. Similarly, they were also the most influential features for predictions on the hidden test set, indicating good generalization. SHAP: SHapley Additive exPlanations. AAC: average acoustic concentration. MBF: mid-band fit. SS: spectral slope. SI: spectral intercept. GLCM: gray-level co-occurrence matrix. GLSZM: gray-level size zone matrix. GLDM: gray-level dependence matrix. NGTDM: neighboring gray tone difference matrix. GL: gray-level, RMAD: robust mean absolute deviation.

We utilized SHapley Additive exPlanations (SHAP) analysis to rank the features based on their importance to the predictions [27]. We utilized the SHAP python package (v. 0.49.1) to perform the analysis and generate visualizations [27]. Figure 4 displays bar plots of the mean absolute SHAP value for each feature in the thirteen-feature model across the validation and test sets. The most important features for the predictions were found to be consistent across the five-CV folds on the development set. In particular, the first-order statistical variance from the margin MBF map (1.11 ± 0.19), the 2D shape feature elongation (0.72 ± 0.06), and the GLCM inverse variance from the core MBF map (0.51 ± 0.15) were consistently identified as the most influential features, where the average and standard deviation of mean absolute SHAP values across CV folds are provided in parentheses. On the hidden test set, these three features remained the most important for the predictions.

## 4. Discussion

The high incidence and morbidity rate associated with breast cancers underscore the importance of early detection, where timely interventions or treatments can significantly improve prognosis. While screening mammography serves as a widely available screening modality, the use of ionizing radiation and potential variability in mammogram interpretation present opportunities for developing a more robust diagnostic framework. The approach used here is grounded in domain expert knowledge which involves extracting radiomics features from QUS spectral parametric images. First-order statistical and textural features of these images provide insights into the distribution of acoustic scatterers and their heterogeneity assessment, enabling the differentiation between malignant and benign breast tumors. Utilizing a subset of these discriminative features, a multivariate diagnostic model was trained to characterize breast lesions. The approach implemented here demonstrates strong generalization. A thirteen-feature model achieved a maximum generalization performance with 92% recall, 89% specificity, 91% balanced accuracy, and a 0.97 AUROC on an independent test set. Furthermore, thirteen-feature models achieved an average recall of 82 ± 8%, average specificity of 79 ± 9%, average balanced accuracy of 80 ± 5%, and an average AUROC of 0.87 ± 0.05 on hidden test sets. Offering a robust, objective, non-ionizing, and portable means for breast cancer screening, the QUS radiomics framework developed here holds significant promise.

In a study by Sadeghi-Naini et al., malignant and benign breast lesions were identified in a cohort of *n* = 78 participants [10]. That work developed classification models using first-order statistical means and GLCM-based textures from QUS spectral parametric images [10]. Osapoetra et al. refined the analysis by incorporating texture-derivative features, hypothesized to be more robust than average texture measures, providing local quantification of image textures. Additionally, that work expanded the cohort to *n* = 204 participants [11]. However, those studies were confined to textural quantification solely through GLCM textures. To address this limitation, Osapoetra et al. investigated the effect of different methods for quantifying image textures on the accuracy of breast lesion classification [12].

The current study advances prior research [10,11,12] by enhancing model-building and evaluation strategies, standardizing the feature extraction process, evaluating a broader range of machine learning classifiers, and expanding the cohort size (*n* = 277). In the work here, we addressed the critical issue of data leakage in machine learning classification model development. Specifically, we partitioned the dataset into development and test folds prior to further analysis, ensuring the validity of the generalization assessment for our proposed approach. The feature extraction procedure was standardized using the PyRadiomics library [26]. Subsequently, data preprocessing steps were applied, including skew correction, outlier detection, feature standardization, feature selection, and data balancing. Features that are normally distributed tend to be useful when building a model, as they exhibit substantial variation for discriminating between classes [47]. Such features were obtained through a skew correction procedure. Furthermore, feature standardization is crucial to prevent certain features with relatively large values from dominating the analysis. Subsequently, both filter-based and wrapper-based techniques were employed to select the optimal set of features. Finally, the training dataset was balanced before building the model.

Among the selected features for the 13-feature SVM-Linear model, the majority (nine features) were first-order and textural features from the MBF parametric image. MBF parametric maps depict the spatial distribution of the amount of acoustic backscattering from tumors. This spectral parameter is sensitive to distinct microstructures of malignant and benign breast lesions. Previous studies have demonstrated the utility of this parameter for characterization and response prediction [10,11,12,15,16,17,18]. Therefore, it is expected that first-order and second-order statistical assessments of the MBF spectral parameter provide robust features for distinguishing malignant from benign breast lesions.

In this study, averaged across 50 partitions, the SVM-Linear achieved a recall of 82 ± 8%, specificity of 79 ± 9%, and an AUROC of 0.87 ± 0.05. Similarly, the LDA classifier resulted in a recall of 80 ± 8%, specificity of 78 ± 8, and an AUROC of 0.87 ± 0.05. On an independent test set, SVM-Linear achieved a peak performance of 92% recall, 89% specificity, and 0.97 AUROC. LDA produced comparable results with 95% recall, 89% specificity, and 0.97 AUROC. Furthermore, considering the importance of accounting for sources of randomness in the design of machine learning experiments [48], model building and evaluation were repeated on fifty randomly stratified splits of the whole dataset. These results indicate that linear models, particularly SVM-Linear and LDA, generalized well for this dataset. Notably, the balanced accuracy scores for these classifiers were statistically significantly better (*p* < 0.05) than those of the other classifiers we compared.

Recently, Destrempes et al. conducted a study aimed at classifying malignant from benign solid breast lesions in a cohort of 103 women with suspicious breast lesions [13]. They developed classification models using various combinations of shear wave elasticity, spectral backscatter, US RF envelope statistics features, and the BI-RADS Score [13]. In their study, the random forest classifier achieved the best classification result with an AUROC of 0.97 and a specificity of 75.9% at 98% sensitivity [13]. Subsequently, Chowdhury et al. reported a model for the classification of breast masses using radiomics features from US Nakagami parametric images [21]. In a cohort of 130 participants with breast lesions (104 benign and 26 malignant), they reported a maximum classification performance of 100% sensitivity, 91.3% specificity, and 93.1% accuracy [21]. In contrast with Destrempes et al.’s work, the multi-variate classification model in the study here only incorporates QUS spectral features and their texture parameters. Destrempes et al.’s model included a clinical feature in BI-RADS score to achieve a 0.97 classification AUROC. Without the BI-RADS score, they reported a 0.74 classification AUROC, based on QUS spectral and shear wave elastography (SWE) features [13]. Furthermore, Chowdhury et al. only reported classification performance on the validation sets. The lack of an actual test set limits the generalization assessment of their proposed model [21].

We conducted a sensitivity analysis to investigate different data-centric approaches for handling data imbalance. In particular, we compared the SMOTE with the RUS approach. RUS resulted in optimal generalization performance, achieving 81 ± 7% recall, 80 ± 9% specificity, and 0.87 ± 0.06 AUROC for the LDA model. Similarly, the SVM-RBF achieved 80 ± 8% recall, 82 ± 8% specificity, and 0.87 ± 0.05 AUROC. These RUS-based results are comparable to the SMOTE-based test performances. Although there was no statistically significant difference in the peak performance between the two approaches to handling data imbalance, the RUS-based approach is evidently more robust in handling the minority class for most classifiers, as indicated in the higher average test specificity metric.

Comparison with the baseline standard obtained from thresholding the BI-RADS categories indicated that the QUS radiomics and machine learning framework obtained better specificity and balanced accuracy (*p*-values < 0.05) on the suspicious breast lesions. The SVM-Linear model achieved an average test performance of 74 ± 18% recall, 78 ± 19% specificity, and 76 ± 12% balanced accuracy. On the other hand, thresholding the BI-RADS resulted in an average test performance of 98 ± 5% recall, 39 ± 26% specificity, and 68 ± 13% balanced accuracy. The proposed framework, in conjunction with the BI-RADS, can potentially mitigate the unnecessary biopsies resulting from the low specificity of the BI-RADS, especially for the suspicious breast lesion cohort (BI-RADS category 4). The choice of BI-RADS ≥ 4 as the threshold for malignancy was driven by the limited availability of the more detailed BI-RADS 4 subcategories (4A, 4B, and 4C) in the clinical patient history.

One morphological feature, elongation, emerged as one of the most selected features. Elongation describes the ratio between the two largest principal components in tumor shape, measuring the axes’ length of the ROI-enclosing ellipsoid [26]. The ratio was observed to be discriminative between malignant and benign breast lesions. This can be attributed to differences in the ROI sizes and general morphological characteristics of the tumors, as previously indicated by Stavros et al. They highlighted that malignant lesions typically exhibited a “taller than wide” characteristic [7], contrasting with the more ellipsoidal shape of benign lesions. The elongation feature captures this distinction of the principal component axes and their ratio. Given that the elongation feature can be interpreted as a quasi-clinical feature, we conducted further analysis to assess the impact of removing morphological features from the pool of radiomics features. The SVM-Linear model, based solely on first-order statistics and textural features, remained robust and achieved the maximum classification performance of 84% recall, 89% specificity, 87% balanced accuracy, and 0.94 AUROC on an independent test set. Additionally, it resulted in an average test performance of 78 ± 8% recall, 74 ± 10% specificity, 77 ± 5% balanced accuracy, and 0.84 ± 0.06 AUROC.

Beyond analyzing classification performance, we employed SHapley Additive exPlanations (SHAP) analysis [27] to rank features according to their contribution to the model’s predictions. The top features driving the predictions were consistently identified across the five-CV folds on the development set. The first-order statistical variance from the margin MBF map, the 2D shape feature elongation, and the GLCM inverse variance from the core MBF map remained the most important predictors in the test set. Apart from the elongation feature that is correlated with tumor size, the predominant features driving the predictions are associated with the heterogeneity of the tumor margin. These differences in the characteristics of the tumor periphery may indicate tumor infiltration in malignant lesions, whereas they reflect benign breast tissue characteristics in benign lesions.

In this study, we applied a traditional machine learning approach. Future work with traditional machine learning approaches can utilize texture-derivative features from intermediary texture maps. Although still limited in their actual interpretations, texture-derivative features have proven to be useful for characterization [11] and for response prediction [16,17]. Currently, we are exploring advanced deep learning architectures, including convolutional neural networks (CNNs) [49] and more recent vision transformers (ViTs) [50], to directly learn useful representations from QUS spectral parametric images. Our most recent work indicated that CNNs significantly outperformed conventional machine learning for this classification task [51]. We anticipate that the ViT approach will also significantly enhance the classification performance.

There are some study limitations in the work here. Due to its single-institutional nature, it is crucial to further validate the proposed framework against data collected from multiple institutions. This effort will be dedicated to future studies utilizing both internal and external validation cohorts to further assess the generalizability of the framework. In addition, we relied on manual segmentation to identify the ROIs for analysis. Although manual contouring is inevitably subject to inter-observer variability, we meticulously performed this step, guided by clinical patient records that provide radiological details and images of the corresponding lesions. Webb et al. quantified the inter-observer variability associated with manual segmentation of breast masses on US images [52]. Their study included three expert annotators (two trained radiologists and one highly expert sonographer; the authors did not report their years of experience) [52]. On a separate test set consisting of 121 images from 85 patients, the average Dice similarity coefficient (DSC) was approximately 0.80, with pairwise Dice scores of 0.814, 0.804, and 0.792 between observers, indicating good agreement among the annotators [52]. These DSCs were comparable with the DSCs reported by Granzier et al. from breast lesions segmentation on MRI images [53]. Granzier et al. reported an average volumetric DSC of 0.81 (range 0.19–0.96) across all tumors, from four annotators of varying expertise. The average DSC for each observer combination ranged from 0.78 and 0.83 [53]. Given the agreements between the annotators, Granzier et al. reported averaged intra-class correlation coefficients (ICCs) greater than or equal to 0.80 for the morphological, first-order statistical, and textural features [53]. Given the similar inter-observer variability observed in the studies by Webb et al. and Granzier et al., we anticipate that a similar feature robustness result can be obtained for radiomic features of QUS spectral parametric images, with respect to inter-observer variability. A separate future work will quantitatively evaluate the impact of manual segmentation on the segmentation accuracy and extend our radiomic framework by incorporating deep neural networks to enable more automated and objective ROI annotation. Lastly, the potential heterogeneity in the reference standard due to benign lesions confirmed solely through imaging surveillance could be alleviated in future studies by including only benign lesions with histopathological confirmation.

## 5. Conclusions

To conclude, this study demonstrates the robustness of the QUS radiomics framework for characterizing breast lesions, as evaluated on a larger patient cohort. The conventional machine learning approach, utilizing comprehensive handcrafted radiomics features from QUS spectral parametric images, resulted in a robust classification model. The analysis here followed strict guidelines for model building and evaluation, allowing for a proper estimation of model’s generalization performance. The results from our expanded dataset demonstrate the potential of the QUS radiomics framework and support the need for future validation studies to support its implementation as a working tool for rapid breast cancer screening in clinical settings.

## Figures and Tables

**Table 1:** Clinical characteristics of the breast characterization cohort.

Characteristic	Benign (*n* = 92)	Malignant (*n* = 185)
Age (y)		
Mean (SD)	44 (12)	53 (12)
Median (Q1, Q3)	46 (38, 51)	51 (44, 63)
Min, max	18, 75	27, 81
Tumor size (cm)		
Mean (SD)	1.8 (1.2)	3.6 (2.0)
Median (Q1, Q3)	1.5 (1.1, 2.4)	3.1 (2.2, 4.5)
Min, max	0.4, 9.6	0.6, 12.0
Non-invasive tumor type		
	FA (*n* = 51)	
	Simple Cyst (*n* = 20)	
	Complicated Cyst (*n* = 11)	
	Benign other (*n* = 10)	
Invasive tumor type		
		IDC (*n* = 168)
		ILC (*n* = 9)
		Malignant other (*n* = 8)

Benign others include lactating adenoma (*n* = 1), PASH (*n =* 4), sclerosing adenosis, stromal fibrosis (*n* = 1), no specific (*n =* 3), and benign breast tissue with calcifications in benign ducts (*n* = 1). Malignant others include invasive metaplastic carcinoma (*n* = 1), poorly differentiated malignant neoplasm (*n* = 1), invasive mammary carcinoma with metaplastic features (*n* = 1), DCIS (*n* = 3), invasive breast carcinoma of no special type (*n =* 1), and invasive breast carcinoma with micropapillary features (*n* = 1). FA: Fibroadenoma. PASH: Pseudo-angiomatous stromal hyperplasia. IDC: Invasive ductal carcinoma. ILC: Invasive lobular carcinoma. DCIS: Ductal carcinoma in situ.

**Table 2:** Test set classification performance. Comprehensive summary of classification performance evaluated on the hidden test sets. Classification metrics include recall, specificity, accuracy, balanced accuracy, precision, NPV, F1-Score, and AUROC. For each classifier, score types include both the average performance from 50 partitions (Average) and the best single partition performance (Best), based on the AUROC metric. The 95% CIs were provided for each metric; these were derived from bootstrapping test scores for all 50 partitions. Across the evaluated machine learning models, linear classifiers in the SVM-Linear and the LDA generalized with the best accuracy and balanced accuracy scores ~80%.

Classifier	Score Type	Recall [95% CI]	Specificity [95% CI]	Accuracy [95% CI]	Balanced Accuracy [95% CI]	Precision [95% CI]	NPV [95% CI]	F1-Score [95% CI]	AUROC [95% CI]	AUPRC [95% CI]
**LDA**	Average	80 ± 8 [58–97]	78 ± 8 [50–100]	79 ± 5 [64–93]	79 ± 5 [63–92]	88 ± 4 [73–100]	69 ± 8 [43–92]	83 ± 5 [68–94]	0.87 ± 0.05 [0.68–0.98]	0.92 ± 0.04 [0.77–0.99]
	Best	95	89	93	92	95	89	89	0.97	0.99
**KNN *k* = 1**	Average	79 ± 8 [56–96]	60 ± 10 [28–88]	72 ± 6 [54–86]	69 ± 6 [50–85]	79 ± 4 [62–93]	60 ± 10 [30–89]	79 ± 5 [62–90]	0.69 ± 0.06 [0.50–0.85]	0.77 ± 0.04 [0.61–0.90]
	Best	89	84	88	87	92	80	82	0.87	0.89
**SVM-Linear**	Average	82 ± 8 [59–97]	79 ± 9 [50–100]	81 ± 5 [64–93]	80 ± 5 [64–93]	88 ± 4 [74–100]	70 ± 9 [43–94]	85 ± 4 [69–95]	0.87 ± 0.05 [0.71–0.98]	0.92 ± 0.03 [0.80–0.99]
	Best	92	89	91	91	94	85	87	0.97	0.99
**SVM-RBF**	Average	84 ± 7 [58–97]	54 ± 20 [27–95]	74 ± 6 [59–89]	69 ± 9 [51–89]	79 ± 7 [65–97]	63 ± 15 [30–91]	81 ± 5 [66–93]	0.80 ± 0.07 [0.65–0.96]	0.89 ± 0.05 [0.77–0.98]
	Best	95	68	86	82	85	87	76	0.94	0.98
**AdaBoost**	Average	81 ± 7 [60–97]	67 ± 11 [36–94]	76 ± 6 [59–91]	74 ± 7 [56–90]	83 ± 5 [67–97]	65 ± 9 [38–92]	82 ± 5 [67–93]	0.82 ± 0.05 [0.63–0.95]	0.89 ± 0.04 [0.75–0.98]
	Best	95	74	88	84	88	88	80	0.92	0.97
**Gradient Boosting**	Average	82 ± 7 [59–97]	68 ± 11 [33–95]	77 ± 6 [61–91]	75 ± 6 [56–91]	84 ± 5 [67–97]	66 ± 9 [38–93]	82 ± 5 [67–94]	0.84 ± 0.05 [0.66–0.96]	0.91 ± 0.03 [0.79–0.98]
	Best	92	74	86	83	87	82	78	0.94	0.97
**Shallow MLP**	Average	82 ± 8 [58–100]	63 ± 13 [26–95]	76 ± 6 [55–91]	73 ± 7 [50–91]	82 ± 5 [63–97]	65 ± 10 [31–100]	82 ± 5 [63–94]	0.81 ± 0.07 [0.59–0.98]	0.88 ± 0.06 [0.73–0.99]
	Best	89	79	86	84	89	79	79	0.94	0.97

LDA: linear discriminant analysis. NPV: negative predictive value. AUROC: area under the receiver operating characteristic curve. AUPRC: area under the precision–recall curve. CI: confidence interval.

**Table 3:** Optimum feature set. The most selected thirteen features for LDA and SVM-Linear models across fifty random splits of the dataset. The selected features include first-order statistics, morphological, and textural features from both tumor core and margin, predominantly from the MBF maps.

#	LDA	SVM-Linear
1	Elongation	Elongation
2	Margin SS-NGTDM Coarseness	Margin AAC-NGTDM Coarseness
3	Margin AAC-NGTDM Coarseness	Margin SS-NGTDM Coarseness
4	Core SI-GLCM Maximum Probability	Core MBF-GLCM Maximum Probability
5	Core MBF-GLCM Maximum Probability	Margin MBF-GLSZM Large Area Low Gray-Level Emphasis
6	Core ASD-GLCM Sum Entropy	Margin MBF First-Order 90th Percentile
7	Core MBF First-Order Uniformity	Margin MBF First-Order Mean Absolute Deviation
8	Margin MBF First-Order 90th Percentile	Core MBF-GLCM Joint Entropy
9	Margin MBF First-Order Variance	Core MBF First-Order Uniformity
10	Margin AAC First-Order Minimum	Margin MBF First-Order Variance
11	Margin MBF-NGTDM Busyness	Margin MBF-NGTDM Busyness
12	Margin SI-GLCM Joint Entropy	Core MBF-GLCM Sum Entropy
13	Core ASD-NGTDM Busyness	Core SI-GLCM Maximum Probability

ASD: Average scattering diameter. AAC: average acoustic concentration. MBF: mid-band fit. SS: spectral slope. SI: spectral intercept. GLCM: gray-level co-occurrence matrix. GLSZM: gray-level size zone matrix. NGTDM: neighboring gray tone difference matrix.

**Table 4:** Sensitivity analysis of approaches to data imbalance on the generalization performance. Models developed using RUS showed recall comparable to SMOTE (Table 2), while improving specificity for the minority benign group across classifiers. This suggests that RUS may provide better class balancing for this dataset and task. The 95% CIs were derived by bootstrapping test scores across all 50 partitions. For each classifier, score types include both the average performance from 50 partitions (Average) and the best single partition performance (Best), based on the AUROC metric.

Classifier	Score Type	Recall [95% CI]	Specificity [95% CI]	Accuracy [95% CI]	Balanced Accuracy [95% CI]	Precision [95% CI]	NPV [95% CI]	F1-Score [95% CI]	AUROC [95% CI]	AUPRC [95% CI]
**LDA**	Average	81 ± 7 [58–95]	80 ± 9 [47–100]	81 ± 6 [63–91]	81 ± 6 [61–92]	89 ± 5 [71–100]	69 ± 9 [41–90]	74 ± 7 [68–94]	0.87 ± 0.06 [67–97]	0.93 ± 0.05 [77–99]
	Best	89	89	89	89	94	81	85	0.97	0.99
**KNN *k* = 1**	Average	76 ± 7 [50–90]	69 ± 11 [35–94]	74 ± 5 [55–88]	73 ± 6 [52–87]	83 ± 5 [64–97]	60 ± 7 [32–81]	64 ± 7 [60–90]	0.73 ± 0.06 [52–87]	0.79 ± 0.04 [62–91]
	Best	78	89	82	84	94	68	77	0.84	0.88
**SVM-Linear**	Average	79 ± 8 [54–93]	80 ± 9 [48–100]	79 ± 6 [61–89]	79 ± 6 [59–91]	89 ± 5 [71–100]	67 ± 8 [38–86]	72 ± 7 [65–93]	0.87 ± 0.05 [67–97]	0.92 ± 0.04 [76–99]
	Best	92	89	91	91	94	85	87	0.96	0.98
**SVM-RBF**	Average	80 ± 8 [49–93]	82 ± 8 [50–100]	80 ± 5 [59–91]	81 ± 5 [61–92]	90 ± 4 [71–100]	68 ± 9 [38–86]	74 ± 6 [62–93]	0.87 ± 0.05 [66–96]	0.93 ± 0.03 [78–98]
	Best	95	84	91	89	92	89	86	0.97	0.99
**AdaBoost**	Average	76 ± 8 [50–93]	72 ± 9 [41–95]	75 ± 6 [55–88]	74 ± 6 [55–88]	84 ± 5 [67–97]	62 ± 9 [35–86]	66 ± 7 [60–91]	0.82 ± 0.06 [61–95]	0.90 ± 0.04 [73–98]
	Best	86	84	86	85	91	76	80	0.91	0.95
**Gradient Boosting**	Average	77 ± 8 [53–94]	77 ± 8 [45–100]	77 ± 5 [59–91]	77 ± 5 [58–92]	87 ± 4 [69–100]	64 ± 8 [38–88]	69 ± 6 [63–93]	0.85 ± 0.04 [68–96]	0.92 ± 0.03 [79–98]
	Best	89	79	86	84	89	79	79	0.92	0.96
**Shallow MLP**	Average	75 ± 13 [51–95]	79 ± 11 [45–100]	77 ± 8 [59–91]	77 ± 7 [60–92]	88 ± 5 [70–100]	64 ± 10 [38–89]	70 ± 8 [63–93]	0.85 ± 0.06 [65–97]	0.91 ± 0.05 [75–99]
	Best	81	95	86	88	97	72	82	0.94	0.97

SMOTE: synthetic minority oversampling technique. RUS: random undersampling. NPV: negative predictive value. AUROC: area under the receiver operating characteristic curve. AUPRC: area under the precision–recall curve. CI: confidence interval.

**Table 5:** Comparison of test set classification performance between QUS radiomics and BI-RADS. We compared the classification performance from the best model with the baseline standard of BI-RADS categories, thresholded at BI-RADS category 4. The SVM-Linear model attained significantly higher (*p*-values < 0.05) average test specificity and balanced accuracy than those from the BI-RADS.

Classifier	Recall	Specificity	Accuracy	Balanced Accuracy	Precision	NPV	F1-Score	AUROC	AUPRC
**BI-RADS**	98 ± 5	39 ± 26	73 ± 15	68 ± 13	70 ± 17	82 ± 34	80 ± 13	N/A	N/A
**QUS Radiomics**	74 ± 18	78 ± 19 (*)	75 ± 10	76 ± 12 (*)	81 ± 21	69 ± 17	76 ± 16	0.84 ± 0.11	0.90 ± 0.09

Statistically significant metrics are indicated by the asterisks (*). BI-RADS: breast imaging reporting and data system. SVM: support vector machine. NPV: negative predictive value. AUROC: area under the receiver operating characteristic curve. AUPRC: area under the precision–recall curve.

**Figure A1:**
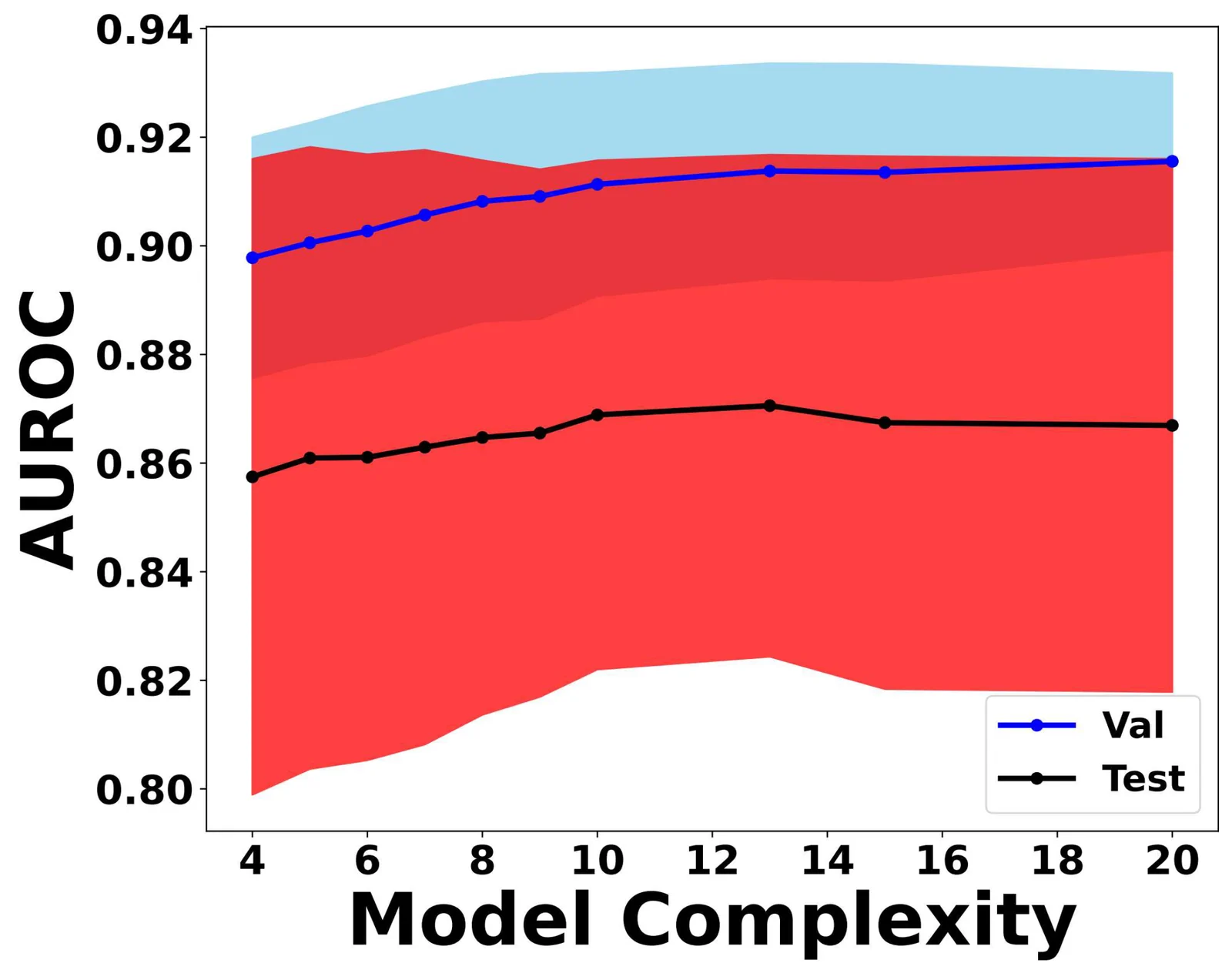
**Model complexity analysis.** AUROC versus feature number for the validation and test sets for the SVM-Linear models. The number of selected features was varied including {4, 5, 6, 7, 8, 9, 10, 13, 15, and 20}. The average AUROCs, along with ± standard deviations, across the 50 partitions are shown for the validation and test sets, respectively. Validation performance is indicated by the solid blue line (average), with its range indicated by the light-blue shade. Test performance is indicated by the solid black line, with its range indicated by the red shade. The average generalization performance (on independent test sets) peaked at the 13-feature model and subsequently slightly decreased as the complexity is increased. The validation performance (which is part of the model development) keeps increasing as the complexity increases.

## Data Availability

The datasets used and/or analyzed during the current study are available from the corresponding author on reasonable request.

## Abbreviations

The following abbreviations are used in this manuscript:

QUS	Quantitative ultrasound
SVM	Support vector machine
IQR	Inter-quartile range
AUROC	Area under the receiver operating characteristic curve
AUPRC	Area under the precision–recall curve
DCE-MRI	Dynamic contrast-enhanced magnetic resonance imaging
US RF	Ultrasound radio-frequency
SWE	Shear wave elastography
GLCM	Gray-level co-occurrence matrix
GLRM	Gray-level run-length matrix
GLSZM	Gray-level size zone matrix
SHAP	SHapley Additive exPlanations
BI-RADS	Breast imaging reporting and data system
MBF	Mid-band fit
SS	Spectral slope
SI	Spectral intercept
BSC	Backscatter coefficient
ASD	Average scattering diameter
AAC	Average acoustic concentration
NGTDM	Neighboring gray tone difference matrix
GLDM	Gray-level dependence matrix
CV	Cross-validation
MRMR	Maximal relevance minimal redundancy
SMOTE	Synthetic minority oversampling technique
RUS	Random undersampling
SFS	Sequential feature selection
LDA	Linear discriminant analysis
KNN	*k*-nearest neighbors
SVM-RBF	Support vector machine-radial basis function
AdaBoost	Adaptive boosting
MLP	Multilayer perceptron
PPV	Positive predictive value
NPV	Negative predictive value
FA	Fibroadenoma
PASH	Pseudo-angiomatous stromal hyperplasia
IDC	Invasive ductal carcinoma
ILC	Invasive lobular carcinoma
DCIS	Ductal carcinoma in situ
IMC	Invasive mammary carcinoma
FOV	Field-of-view
IDM	Inverse difference moment
CNN	Convolutional neural network
VIT	Vision transformer
DSC	Dice similarity coefficient
ICC	Intra-class correlation coefficient

## Appendix A

**Table A1:** **US system parameters.** Physical parameters and acoustic beam characteristics from L14-5/60 transducer on a Sonix Touch US system [15].

Parameters	Values
Number of elements	128
Kerf width [µm]	25
Element width [µm]	477
Elevation [mm]	4
Elevation focus [mm]	14
Depth of focus [mm]	16.7
Center frequency [MHz]	6.5
Bandwidth [MHz]	3–8
f\#	1.82
Axial resolution at 15 mm (−6 dB) [µm]	198
Lateral resolution at 15 mm (−6 dB) [µm]	483

**Table A2:** **Radiomics features.** GLCM: gray-level co-occurrence matrix, GRLM: gray-level run-length matrix, GLSZM: gray-level size-zone matrix, GLDM: gray-level dependence matrix, NGTDM: neighboring gray-tone difference matrix.

Morphological Features (*n* = 9)	{ Mesh Surface, Pixel Surface, Perimeter, Perimeter to Surface Ratio, Sphericity, Spherical Disproportion, Maximum 2D Diameter, Major Axis Length, Minor Axis Length, Elongation }
First-Order Statistical Features (*n* = 18)	{ 10th Percentile, 90th Percentile, Energy, Entropy, Interquartile Range, Kurtosis, Maximum, Mean Absolute Deviation (MAD), Mean, Median, Minimum, Range, Robust Mean Absolute Deviation (rMAD), Root Mean Squared (RMS), Skewness, Total Energy, Uniformity, Variance }
GLCM (*n* = 24)	{ Autocorrelation, Cluster Prominence, Cluster Shade, Cluster Tendency, Contrast, Correlation, Difference Average, Difference Entropy, Difference Variance, Inverse Difference (ID), Inverse Difference Moment (IDM), Inverse Difference Moment Normalized (IDMN), Inverse Difference Normalized (IDN), Informational Measure of Correlation (IMC) 1, Informational Measure of Correlation (IMC) 2, Inverse Variance, Joint Average, Joint Energy, Joint Entropy, Maximal Correlation Coefficient (MCC), Maximum Probability, Sum Average, Sum Entropy, Sum Squares }
GRLM (*n* = 16)	{ Gray Level Nonuniformity, Gray Level Nonuniformity Normalized, Gray Level Variance, High Gray Level Run Emphasis, Long Run Emphasis, Long Run High Gray Level Emphasis, Long Run Low Gray Level Emphasis, Low Gray Level Run Emphasis, Run Entropy, Run Length Nonuniformity, Run Length Nonuniformity Normalized, Run Percentage, Run Variance, Short Run Emphasis, Short Run High Gray Level Emphasis, Short Run Low Gray Level Emphasis }
GLSZM (*n* = 16)	{ Gray Level Nonuniformity, Gray Level Nonuniformity Normalized, Gray Level Variance, High Gray Level Zone Emphasis, Large Area Emphasis, Large Area High Gray Level Emphasis, Large Area Low Gray Level Emphasis, Low Gray Level Zone Emphasis, Size Zone Nonuniformity, Size Zone Nonuniformity Normalized, Small Area Emphasis, Small Area High Gray Level Emphasis, Small Area Low Gray Level Emphasis, Zone Entropy, Zone Percentage, Zone Variance }
GLDM (*n* = 14)	{ Dependence Entropy, Dependence Nonuniformity, Dependence Nonuniformity Normalized, Dependence Variance, Gray Level Nonuniformity, Gray Level Variance, High Gray Level Emphasis, Large Dependence Emphasis, Large Dependence High Gray Level Emphasis, Large Dependence Low Gray Level Emphasis, Low Gray Level Emphasis, Small Dependence Emphasis, Small Dependence High Gray Level Emphasis, Small Dependence Low Gray Level Emphasis }
NGTDM (*n* = 5)	{ Busyness, Coarseness, Complexity, Contrast, Strength }

**Table A3:** **Classifiers’ hyperparameters.** SVM: support vector machine, SVM-RBF: support vector machine–radial basis function, AdaBoost: adaptive Boosting, MLP: multilayer perceptron.

Classifier	Hyperparameters	Values
**SVM-Linear**	C	{1 × 10^−2^, 1 × 10^−^^1^, 1.0, 10.0}
**SVM-RBF**	C γ	{1 × 10^−4^, 1 × 10^−3^, 1 × 10^−2^, 1 × 10^−1^, 1, 10} {1 × 10^−3^, 1 × 10^−2^, 1 × 10^−1^, 1, 10, 100, 1000}
**AdaBoost**	n_estimators learning_rate algorithm	{25, 50, …, 200} {0.1, 0.2, 0.3, 0.5, 1.0} {3, 4}
**Gradient Boosting**	n_estimators	{50, 100, …, 550}
	subsample	{0.5, 0.8, 1.0}
	max_leaf_nodes	{4, 5, 6, 7, 8}
	learning_rate	{1 × 10^−3^, 1 × 10^−2^, 1 × 10^−1^}
**Shallow MLP**	hidden_layer_sizes	{2, 4, …, 14}
	activation	{‘relu’, ‘tanh’, ‘logistic’}
	solver	{‘sgd’, ‘adam’, ‘lbfgs’}
	alpha	{1 × 10^−6^, 1 × 10^−5^, …, 1 × 10^−1^}
	batch_size	{16, 32, 64}
	learning_rate	{‘constant’, ‘invscaling’, ‘adaptive’}
	learning_rate_init	{1 × 10^−5^, 1 × 10^−4^, …, 1 × 10^−1^}
	max_iter	{250, 500, 750}
	momentum	{0.90, 0.95, 0.99}

**Table A4:** (**A**). **Frequency distribution of feature selection for LDA.** We tabulate the most frequently selected features for the thirteen feature LDA classifier, across 50 partitions. Morphological feature in 2D elongation was selected 76% of the time, while textural features from tumor margin in NGTDM coarseness AAC and NGTDM coarseness SS (from ~40% to ~50% of the time). (**B**). **Frequency distribution of feature selection for SVM-Linear.** We tabulate the most frequently selected features for the thirteen-feature SVM-Linear classifier, across 50 partitions.

(**A**)	
**Feature**	**Count [Frequency]**
Elongation	38 [0.76]
NGTDM Coarseness Margin SS	27 [0.54]
NGTDM Coarseness Margin AAC	22 [0.44]
GLCM Maximum Probability Core MBF	14 [0.28]
First-order Uniformity Core MBF	13 [0.26]
GLCM Maximum Probability Core SI	13 [0.26]
First-order Variance Margin MBF	12 [0.24]
GLCM Sum Entropy Core ASD	10 [0.20]
First-order 90th Percentile Margin MBF	9 [0.18]
First-order Minimum Margin AAC	8 [0.16]
GLCM Joint Entropy Margin SS	7 [0.14]
GLCM Joint Energy Core SI	7 [0.14]
GLCM Difference Variance Margin AAC	7 [0.14]
(**B**)	
**Feature**	**Count [Frequency]**
Elongation	43 [0.86]
NGTDM Coarseness Margin AAC	23 [0.46]
NGTDM Coarseness Margin SS	19 [0.38]
GLCM Maximum Probability Core MBF	13 [0.26]
First-order 90th Percentile Margin MBF	11 [0.22]
GLSZM Large-Area Low Gray-Level Emphasis Margin MBF	11 [0.22]
First-order Mean Absolute Deviation Margin MBF	10 [0.20]
GLCM Joint Entropy Core MBF	9 [0.18]
First-order Uniformity Core MBF	9 [0.18]
First-order Variance Margin MBF	8 [0.16]
GLRLM Long Run Low Gray-Level Emphasis Core SS	7 [0.14]
GLRLM Gray-Level Nonuniformity Normalized Core MBF	7 [0.14]
NGTDM Busyness Margin MBF	7 [0.14]

**Table A5:** Representative SHAP values across CV folds: mean absolute SHAP values from a 13-feature SVM-Linear model across the five-CV folds.

Feature	Mean($\left|SHAPValue\right|$)— Average ± Std Across CV Folds
First-Order Variance Margin MBF	1.11 ± 0.19
Elongation	0.72 ± 0.06
GLCM Inverse Variance Core MBF	0.51 ± 0.15
GLSZM Gray-Level Nonuniformity Normalized Margin SS	0.44 ± 0.05
GLSZM Zone Entropy Margin SS	0.35 ± 0.10
GLSZM Zone Entropy Margin MBF	0.33 ± 0.10
GLDM Gray-Level Nonuniformity Margin MBF	0.31 ± 0.07
First-Order Robust Mean Absolute Deviation Margin MBF	0.30 ± 0.21
NGTDM Coarseness Margin AAC	0.14 ± 0.07
GLRLM Short-Run High Gray-Level Emphasis Core SI	0.12 ± 0.10
GLDM Large Dependence Low Gray-Level Emphasis Margin SI	0.11 ± 0.09
First-Order Root Mean Squared Margin MBF	0.05 ± 0.05
GLCM Cluster Prominence Margin MBF	0.04 ± 0.01

**Table A6:** (**A**) Comparison of AUROCs between the LDA model and the other classifiers. (**B**) Comparison of AUROCs between the SVM-Linear model and the other classifiers.

(**A**)	
**Classifier**	**AUROC Difference [95% CI]**
**KNN *k* = 1**	0.19 [0.03–0.30]
**SVM-Linear**	0.00 [−0.08–0.09]
**SVM-RBF**	0.06 [−0.09–0.13]
**AdaBoost**	0.05 [−0.07–0.16]
**Gradient Boosting**	0.02 [−0.07–0.15]
**MLP**	0.05 [−0.10–0.19]
(**B**)	
**Classifier**	**AUROC Difference [95% CI]**
**L DA**	0.00 [−0.08–0.09]
**KNN *k* = 1**	0.19 [−0.06–0.32]
**SVM-RBF**	0.04 [−0.06–0.14]
**AdaBoost**	0.06 [−0.06–0.19]
**Gradient Boosting**	0.03 [−0.09–0.15]
**MLP**	0.04 [−0.06–0.19]

## Appendix B

The computational complexity using repeated hold-out, with a repeat k-fold internal cross-validation is

$$O\left(m\cdot r\cdot k\cdot T\left(n\right)\right)$$

where

m= number of repeat hold-out validations (m= 50);

r= number of repeated internal stratified CVs (r= 3);

k= number of internal CV folds (k= 5);

T(n)= time complexity of training the model on n samples (n= 221 development samples).

**Classifier**	**Computational Complexity** T(n)
LDA	$O\left(n\cdot d^{2}+d^{3}\right)$
SVM-Linear	$O\left(n\cdot d\right)$
SVM-RBF	$O\left(n^{2}\cdot d\right)$ and $O\left(n^{3}\cdot d\right)$
AdaBoost	$O\left(B\cdot n\cdot d\right)$
Gradient Boosting	$O\left(T\cdot n\cdot d\cdot \log n\right)$
Shallow MLP	$O\left(E\cdot n\cdot h\cdot \left(d+c\right)\right)$
n= number of training samples; d= number of training features; B= number of boosting rounds (for AdaBoost); T= number of decision trees (DTs) sequentially trained; E= number of training epochs; h= number of input units; d= number of hidden units; c= number of output units (class targets).	
